# CDSS-RM: a clinical decision support system reference model

**DOI:** 10.1186/s12874-018-0587-6

**Published:** 2018-11-16

**Authors:** Dimitrios Zikos, Nailya DeLellis

**Affiliations:** School of Health Sciences, Central Michigan University, Mt. Pleasant, MI USA

**Keywords:** Clinical decision making, Decision-support systems, Reference model, Systems design, Theoretical framework

## Abstract

Clinical Decision Support Systems (CDSS) provide aid in clinical decision making and therefore need to take into consideration human, data interactions, and cognitive functions of clinical decision makers. The objective of this paper is to introduce a high level reference model that is intended to be used as a foundation to design successful and contextually relevant CDSS systems. The paper begins by introducing the information flow, use, and sharing characteristics in a hospital setting, and then it outlines the referential context for the model, which are clinical decisions in a hospital setting. Important characteristics of the Clinical decision making process include: (i) Temporally ordered steps, each leading to new data, which in turn becomes useful for a new decision, (ii) Feedback loops where acquisition of new data improves certainty and generates new questions to examine, (iii) Combining different kinds of clinical data for decision making, (iv) Reusing the same data in two or more different decisions, and (v) Clinical decisions requiring human cognitive skills and knowledge, to process the available information. These characteristics form the foundation to delineate important considerations of Clinical Decision Support Systems design. The model includes six interacting and interconnected elements, which formulate the high-level reference model (CDSS-RM). These elements are introduced in the form of questions, as considerations, and are examined with the use of illustrated scenario-based and data-driven examples. The six elements /considerations of the reference model are: (i) Do CDSS mimic the cognitive process of clinical decision makers? (ii) Do CDSS provide recommendations with longitudinal insight? (iii) Is the model performance contextually realistic? (iv) Is the ‘Historical Decision’ bias taken into consideration in CDSS design? (v) Do CDSS integrate established clinical standards and protocols? (vi) Do CDSS utilize unstructured data? The CDSS-RM reference model can contribute to optimized design of modeling methodologies, in order to improve response of health systems to clinical decision-making challenges.

## Background

Proper use of clinical information is especially important in an effort to make sound clinical decisions and provide quality health services [1]. A variety of information is combined by healthcare professionals who arrive at clinical conclusions [2]. These decisions rely on information which once acquired, is further processed by healthcare professionals’ cognitive skills, such as in the differential diagnosis [3]. Combining clinical information and the cognitive element is therefore critical to clinical decision making.

One highly emerging focus area of medical informatics is to improve care delivery of in-hospital patients with the development of data-driven, patient-centered decision support systems. Development of such systems is a highly demanding and multidisciplinary task that requires the integration of knowledge from the clinical domain, and decision science to adapt the CDSS to the hospital practice and clinical work flows [4]. Clinical Decision Support Systems (CDSS) provide clinicians with knowledge, intelligently filtered or presented at appropriate times, to enhance health and health care [5], and can be seen as an effective pathway to improve patient safety [6], providing, for instance, alerts for error reduction [7]. Therefore, the information that CDSS provide needs to reflect the decision-making process and the intellectual effort of clinicians in a contextually relevant way. CDSS cannot rely on static, prefabricated ‘in vitro’ methods. Instead, CDSS should make dynamic predictions, allowing interactions with clinicians and taking into consideration the longitudinal nature of health and disease [8]. Designing successful CDSS requires collaboration with domain experts who have knowledge of clinical attributes that are required to be used together, due to treatment or diagnostic criteria.

In recent approaches, such as CONFlexFlow [9], integrating flexible clinical pathways into CDSS was recognized as critical for system success and the means to better understand the clinical context through ontologies, and decide the right rules for a certain activity. During the eighties and early nineties, there was an open debate on how “recent progress in computer-based diagnosis has been encouraging enough to consider the concept of computer diagnosis” [10]. Nowadays, while this is still an open debate, the impressive progress in machine learning and artificial intelligence provides new opportunities for more targeted and accurate clinical predictions and recommendations [11].

There is a wide recognition, in the literature, of the importance of secondary use of health data, for decision support and quality improvements. The American Medical Informatics Association convened a summit that resulted in a white paper laying the groundwork for policy, future research, and a taxonomy of uses, recognizing as a requirement, at a nation level, an infrastructure of policies, standards, and best practices, regarding secondary health data analysis [12]. Other authors also discuss the importance of improved utilization of secondary health data [13]. There is therefore an evident consensus that harnessing secondary data provides to health systems enormous opportunities to improve the quality of care and practice.

Despite this, to the authors’ knowledge, there has not been any effort, so far, to systematically illustrate considerations and approaches specific to the conceptualization of data-driven decision support systems for clinical decision making. Although comprehensive efforts specific to CDSS and clinical decision making have not been published, there are available general purpose theoretical frameworks such as the Google TITE (Time-Interactions-Trends-Events) [14], outlining important components for decision support systems, in general. Peripheral work includes another general-purpose artificial intelligence framework to address challenges in the modern healthcare system [15], serving, as a “simulation environment for exploring various healthcare policies, and payment methodologies, forming the basis for clinical artificial intelligence”. Other efforts include the work of Fox et al. [16] who developed the PROforma method, for specifying clinical guidelines and protocols via graphical notation and a formal knowledge representation language. In their paper, they discuss the need for flexible and well understood knowledge representations which are capable of capturing clinical guidelines and protocols for decision support systems. Another effort is the EON project [17] and focuses on the retrieval and use of clinical guidelines using reasoning systems. Additionally, Greenes outlines aspects of CDS for models and frameworks, summarizing the literature [18]. These aspects include the adaptation of CDSS to hospital workflow, construction of its components, interoperability and data sharing, reasoning considerations, health systems priorities, quality improvement outcomes, and CDS effectiveness evaluation. We recognize that these aspects address several different layers of data, analysis, and decisions, including organizational, interoperability, and modeling aspects.

These priorities, as summarized in the literature, address different levels and concerns of data use and modeling. In our work, the specific focus and contribution is the abstraction layer of the conceptual modeling aspects of CDSS via the introduction of the CDSS-RM (Clinical Decision Support System Reference Model) for Computerized, data-driven CDSS design. The paper starts by defining the context that the CDSS-RM reference model is constructed on. The paper continues by outlining five health data use and exchange properties which are required to be considered during the design of CDSS. Then there are eventually introduced six interrelated elements, built around the data properties previously discussed. These six elements form an illustrative reference model for CDSS. In this paper we define the CDSS-RM context, and classify six elements that formulate the CDSS-RM reference model. The authors are health administration researchers, with nursing background and with health analytics research experience. The main author’s clinical practice exposure in both the US and European Health Systems was foundational for the translation of practical perspectives of decision makers to the presented model.

### Definition of the context for the CDSS-RM

**Fig. 1:**
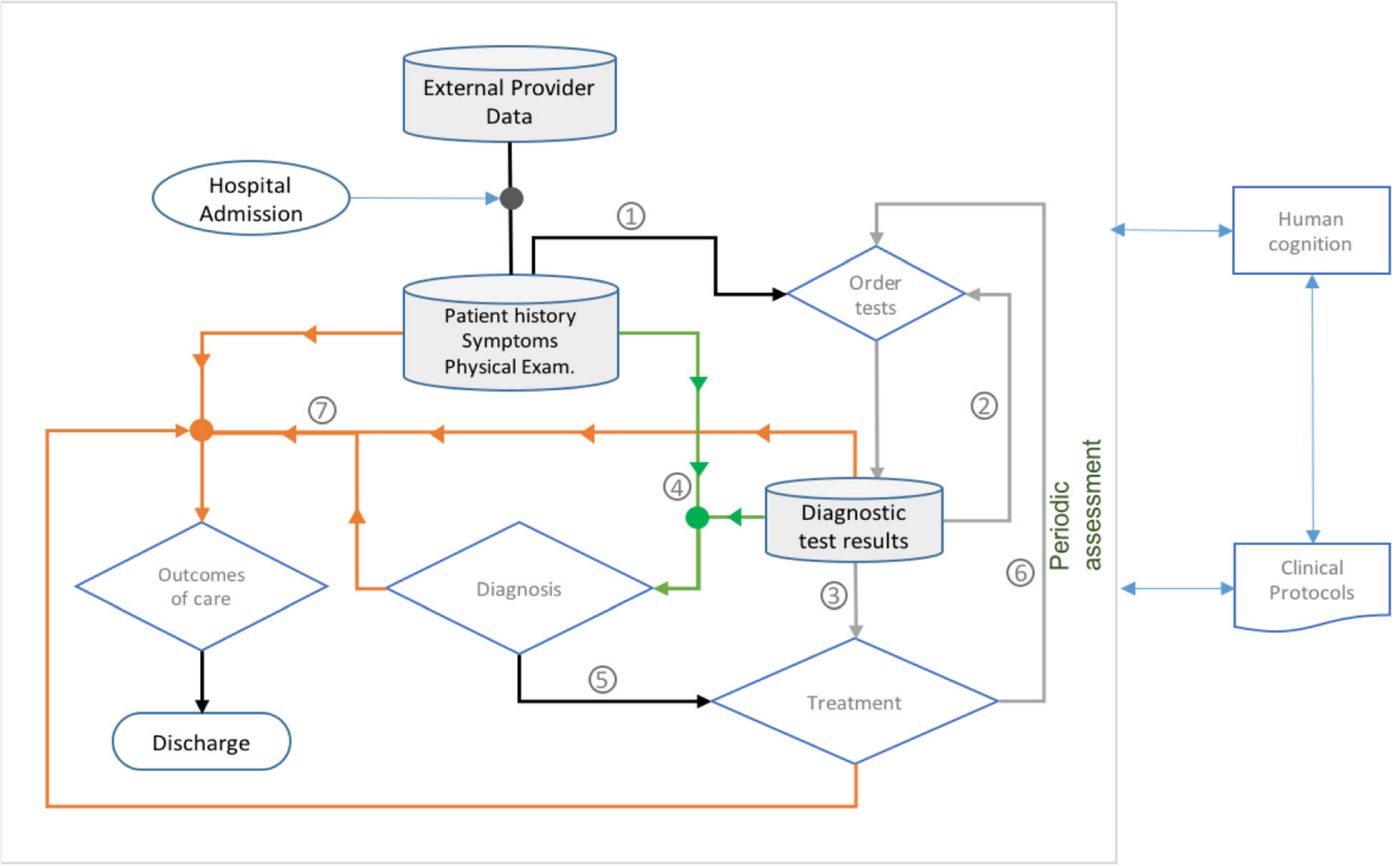
Contextual relevance of the CDSS-RM reference model: The clinical decision-making process

The use context of this reference model is a hospital setting where clinical decision makers make decisions about the patient diagnosis, and treatment. Upon patient admission, the clinical decision process begins at the point shown in Fig. 1. Clinical decisions include the: (i) selection of appropriate diagnostic tests, (ii) patient diagnosis, (iii) selection of optimal treatments and (iv) prediction of the patient prognosis. This is the *decision-making context* that the reference model is based upon. These decisions are interdependent and are characterized from data use and data flow patterns. Below we make an attempt to summarize some of these patterns, as observed in the daily routine hospital practice and as discussed in the literature. Regardless any structural hospital characteristics, and variations found in different health systems, these patterns are inherent to any clinical decision making process. Additionally, in our work, tackling information fragmentation and data-rquality-related challenges of health systems (later presented in this article), belong to a different abstraction layer and therefore not included to the reference model, that intentionally focuses on conceptual modeling elements according to the natural clinical information use and exchange.

1. **Ordered steps, each leading to new data, useful for a new decision:** Patient history, symptoms, and physical examination contribute to decisions for diagnostic test ordering. Test results form the basis for patient diagnosis. The diagnosis, in turn, is decisive for the choice of an optimal treatment. Examples found in the literature demonstrating this workflow include the work of Combi [19]. Additionally, the numerous flowcharts of hospital process analysis that can be found in hospital settings, illustrate this property.
2. **Feedback loops and temporal repetitions:** The result of a diagnostic test may direct physicians to order additional tests, as a requirement for successful differential diagnosis (Fig. 1, point 2). In addition, a diagnostic test may be repeated, during a periodic assessment, to confirm or alter the therapeutic schema in response to the updated diagnostic test results (Fig. 1, point 6). These feedback loops and repeated measurements are often mandated as requirements of hospital clinical pathways and protocols of care. According to Shah [20], evidence and experience in practice should follow a positive feedback loop to construct the decision-making paradigm in patient treatment. Recently, Zikos has presented work of a feedback loop [21] during symptom reporting, where the computer system asks for additional patient information based on their initial reporting, to facilitate improved insights during the triage process, and handle the physician uncertainty.
3. **Combining data:** Naturally, a variety of different data need to be combined in decision making. Examples include combining diagnostic test results with patient history, physical examination and symptoms, to form the diagnosis (Fig. 1, point 4). Or the combination of lab test results, the diagnosis, patient history, physical examination and treatment, to predict the patient prognosis (Fig. 1, point 7).
4. **Decisions made by processing information with cognitive skills and knowledge:** In clinical decisions, clinical data are assessed by health professionals’ knowledge and decision-making skills.
5. **Data reuse:** Clearly, in clinical practice, data facilitate more than one decision. Data generated to support a specific decision-making process, can be reused later on for another decision. For instance, while lab and radiology results are primarily ordered to set a diagnosis, they are later utilized for treatment decisions, or for the patient prognosis.

The focus and attention of the CDSS-RM model is the *conceptual development* of Computerized Clinical Decision Support Systems in hospital care. The model is therefore primarily intended to be useful for Healthcare IT Designers and Consultants, as well as by Healthcare IT Project Managers, to communicate design considerations with health data analysts and IT contractors. Its scope is any in-hospital clinical decision making scenario that requires combination and modeling of clinical data for computer assisted decision support. These decision making procedures include the medical diagnosis, treatment selection, patient prognosis, discharge and patient transfer information, and the selection of appropriate medical procedures. It is therefore relevant as a tool to encapsulate and communicate characteristics that a CDSS may integrate, spanning from the proper selection and modeling of feature-sets, and including high-level approaches for the algorithmic portion of CDSS, instead of the technical algorithmic implementation. The introduction, to the reader, of the reference model is given in a descriptive, rather than prescriptive manner.

### Information use and flow characteristics in healthcare

Clearly, proper use of clinical information is especially important, for high quality health services. Health data properties are delineated extensively in the literature. These include completeness, correctness, concordance, plausibility and currency [22]. Additionally, Hersh et al. also discusses data provenance, granularity, and challenges related to unstructured data. Another classification of data properties, discuss the concept of complete documentation, information breadth, density (no missing time-points), and the predictive strength of input variables [23]. There is quantified evidence of the existence of the aforementioned data challenges, as reported in research, such as in the work of Brotsis et al. [24] who measured data quality issues from Electronic Medical Record Data.

The aforementioned data challenges restrict the potential for successful data modeling. In this work we acknowledge these challenges, however our focus is the design and conceptual modeling considerations, abstracted from data quality issues. Use of data and information flow in healthcare, share fundamental characteristics, which relate to the capacity of clinicians to make good decisions. We classify these properties as: **(1)** Non-atomicity **(2)** Cognition (**3)** Temporality **(4)** Sharing **(5)** Reuse.

#### Non-atomicity

Different segments of health care data should not be assessed independently: Most of the clinically useful information comes by combining multiple data resources and by evaluating combined information with the clinical knowledge of a health professional [25]. Physicians combine the physical examination, laboratory test results and patient history data, for a clinical assessment. A blood glucose measurement of 128 mmHg is evaluated as normal when combined with the patient history of a young Type I diabetes patient, but this would not be the case for a non-diabetic person [26]. The aforementioned inherent need drives a requirement for tools providing to clinicians easy access to patient data and reports, summarizing the available clinical information, at the point of care. While Electronic Health Records automate access to aspects of patient information, to streamline the clinician’s workflow [27], there is oftentimes lack of flexible, problem-specific representations of information to facilitate decision needs.

#### Cognition

Health care data are assessed with cognitive skills of health professionals. Differential diagnosis and other cognitive procedures, are based on critical skill-sets. These knowledge-driven and experiential skills, combined with the clinical information they have at their disposal, drive clinical decisions. This cognitive process is systematic and varies across different healthcare professionals. For example, physicians perform differential diagnosis to differentiate between two or more conditions that share similar signs or symptoms [28]. Medical education and continuing professional development are important success factors for this dimension [29].

#### Temporality

Healthcare data should be assessed with a longitudinal insight [30]. Many clinical procedures are repeated during a patient hospitalization (e.g. vital signs, blood tests) [31]. When these data are reviewed, clinicians recognize temporal patterns and assess the disease progression and treatment effectiveness. Morning blood glucose levels of 135 mmHg would seem elevated for some patient, but the clinician would not be alerted if this value had been lower than previous measurements of that patient. Longitudinal data can form the foundation for predictive modeling of patient outcomes and effectiveness of medical treatments [32].

#### Sharing

Healthcare data are shared across the healthcare system and between health professionals, following medical logic [33]. The importance of shared decision making in health care has been increasingly recognized as important research topic [34]. Clinicians do not act in an introverted manner within the healthcare system. An MRI test cannot be solely assessed by the radiologist; a physician would review the MRI to make informed decisions about the patient. An interoperable environment is required to seamlessly share data. In addition, interprofessional collaboration is critical; health curricula and continuous education programs contribute to developing this competence [35].

#### Reuse

Healthcare data are used in a variety of different clinical decisions. Obviously, a lab test result will drive a medical diagnosis, but it can also be used for the treatment effectiveness evaluation or disease progression. Most importantly, since reuse of clinical data is recognized as essential for improved healthcare management, reduced healthcare costs, and effective clinical research [36], researchers explore ways that clinical attributes of care can contribute beyond driving decisions for individual patients, for a better understanding of the system performance for organizational and quality improvements.

### Elements of the CDSS-RM reference model

In response to the challenges for successful clinical decision making, designers of CDSS and data scientists are required to understand and consolidate the aforementioned data properties. This section introduces six elements critical to the design of CDSS. These elements are presented to the reader in the form of core considerations to be made during design of CDSS. The elements do not dictate the technical implementation details and focus on conceptual development principles of CDSS to construct the CDSS-RM reference model [37].

### Expert systems and other machine learning methods simulating clinicians’ decision making

#### Core consideration 1: Do CDSS mimic the cognitive process of clinical decision makers?

There is a reasonable argument around the importance of systems simulating the human decision-making process. Researchers have identified, decades ago, the need to move to a direction where the human reasoning and judgement could be automated. In the work of Lusted, for instance, back in 1968, it was discussed that greater understanding of human judgment processes involved in diagnosis may enable the investigator to produce these processes more exactly on a computer [38].

### Consideration 1.1: Do the CDSS utilize feedback loops to mimic clinical assessment?

In clinical decisions, successful combination of the clinician cognition with the available clinical information can be of great value. Clinical reasoning approaches and methods have been discussed in the literature [39]. The physician often requests additional information (e.g. more examinations and radiology tests) to assign a diagnosis or decide a treatment; these data provide better input for a complete and successful differential diagnosis [40]. Human diagnostic problem solving has been discussed in the literature, in a domain independent manner [41] as well as far as the disease diagnosis problem is concerned [42]. During the clinical cognitive process, the physician will try to ‘fill in reasoning gaps’ by reassessing the existing information or by ordering more clinical tests. This loop *(…clinician’s assessment ➔ clinical data ➔ clinician’s assessment…)* is a foundational element of clinical decisions. The feedback loop aims to replicate this possible initial de facto clinician uncertainty. CDSS design approaches can simulate the aforementioned loop, by applying reinforcement learning methods [43, 44]. Oftentimes, the probabilistic nature of health and disease results in significant amounts of inappropriate care [45]. The design therefore needs to take this into account by recognizing, and thereafter evaluating other probable factors, to reduce decision uncertainty. The dynamic user feedback loop approaches and reinforcement learning methods have been shown to positively contribute to this direction. The clinical scenario below illustrates how an informed feedback from an algorithm can lead to updated user input, and finally to improved clinician certainty.

Input of symptoms: {x_1_…x_n_} (initial clinical information) ➔ Output (prediction): Probability for Condition A: 80%, Condition B: 65% ➔ Initial model finds that an unreported symptom x_k_ is often present in Conditions A and B ➔ Clinician now requests new information and indeed validates the existence of x_k_ ➔ Initial input is updated as {x_1_…x_n_, x_k_} ➔ New Probabilities calculated: Condition A = 95%, Condition B = 35% ➔ Minimized clinical uncertainty.

### Consideration 1.2: Do CDSS utilize in unison expert systems and machine learning?

Expert systems in healthcare settings are knowledge based systems that imitate the cognitive process of decision making, by using reasoning approaches. Expert systems solve complex problems by reasoning about knowledge, represented mainly in the form of conditional (If-Then-Else) rules [46, 47]. In a clinical setting, there are too many considerations and small but non-trivial clinical details. This is one reason why clinical expert systems have a limited focus on a specific, very well-defined decision-making domain, such as the diagnosis of a given condition. The existence of the more traditional knowledge-based systems (rule-based mimicking of human reasoning) and the more current data driven machine learning algorithms can be complementing to each other. Both technologies are utilized in healthcare settings the common goal to assist clinical decision makers. In medical diagnostic reasoning, there are sometimes patient cases where the compiled knowledge fails to recognize a condition: This is an evident limitation when conditions appear in unexpected or unusual manners and when some patients manifest rare findings or disorders. The aforementioned limitation can be handled with modeling of enormous historical clinical datasets, sufficient in size to include patters of disease for such rare and unique cases. By marrying expert system approaches, which mimic reasoning in decision making, with machine learning methods, this common objective can be met with higher success [48].

### The temporal nature of clinical decision making

#### Core consideration 2: Do CDSS provide recommendations with longitudinal insight?

When healthcare professionals review patient information, they typically compare physiological measurements and laboratory results against physiological norms [49]. Physicians do not just review raw physiological measurements, but they primarily want to know how the patient responds to their therapy of choice, by anticipating improved physiological measurements. Clinical decision makers also consider, during patient assessment, what physiological values they would expect, given the patient response to the therapy. Physiological values, when compared against recent measurements and baselines for the patient under investigation, provide improved insights about treatment effectiveness or disease progression. This section explains three CDSS design considerations related to this aspect: a) Inclusion, as predictors, trends of repeated measurements (b) Modeling of the sequential order of clinical events (c) Modeling of the temporal distance between clinical events.

### Consideration 2.1. Do CDSS use trends of physiological measurements instead of cross-sectional data?

Evidently, use of cross-sectional data does not allow assessment of longitudinal care, which may be more important than visit-based indicators [50]. For some patient, blood glucose levels of 150 mg/dl, might not raise concerns, provided that the glucose levels for that patient were 180 mg/dl in the previous day, and 210 mg/dl two days before. Clearly, despite the increased value of 150 mg/dl, the physician observed satisfactory response to therapy and would not alter the therapeutic schema. For a second patient, though, a blood glucose measurement of 150 mg/dl, would lead to a different clinical decision if this measurement was the only known one for that second patient. This case, would require the physician’s attention. In those two different scenarios, while the cross-sectional input value is the same (blood glucose = 150 mg/dl), the model output clearly depends on previous measurements. This example aims to illustrate the longitudinal nature of clinical decisions and this is why a longitudinal medical record is key to clinical decision support [51]. Temporal trends and fluctuating results of repeated physiological measurements should be significant considerations when designing CDSS.

To further elaborate, we generated a dataset 200 chronic diabetes patients who were admitted with pneumonia (J18.9 ICD-10-CM), and, as a result, uncontrolled diabetes (E11.65 ICD-10-CM). While this is not a synthetic dataset, we generated values based on the fundamental knowledge that, during hospital admission, for patients with uncontrolled diabetes (i) the higher the blood glucose levels, the longer the stay [52] and (ii) the faster the blood glucose levels are controlled, the shorter the hospital stay. Using an online realistic data generator (mockaroo.com) with the aforementioned criteria as functions, we created the following variables and 200 tuples of data: Consecutive blood glucose measurements per patient, age, sex and length of stay. Using this generated dataset, the variables *{Blood Glucose value, Gender, Age}* were utilized to predict the hospital Length of Stay (LOS), using the Weka data mining software, version 3.8. At first, we estimated the mean blood glucose *(Mean BG)* per patient and used it as input variable, together with the demographics, for the prediction of the *LOS*. Using the Weka implementation of linear regression (Weka LinearRegression function) with the “Enter” variable method, the R squared value was found equal to 0.56, and the absolute error was 67.57%. Then, we generated a new attribute, the *Blood Glucose Trend (BG Trend)*, for the three consecutive blood glucose measurements. The BG Trend variable takes integer values from − 2 to + 2, where 0 indicates stable blood glucose levels, + 2 significant increase, and − 2, is a significant drop. This attribute was added to the feature-set *{Blood Glucose Trend (increase/stability/decrease), Gender, Age}* and another linear regression equation was estimated. This time, the R squared value was significantly higher and equal to 0.84, and the relative absolute error went down to 44.57% (Table 1). Prediction of the *LOS* was evidently more accurate for the second model that considered the temporal progress of the condition [38]. In both models we included the mean BG variable as input, serving as the patient baseline information. We acknowledge the limitation of this data experiment, though, that is the use of simulated data based on researcher defined criteria for data generation.

**Table 1:** Model performance improves when trends and temporal changes are taken into account

Feature set (input)	AGE, SEX, MEAN BG	AGE, SEX, MEAN BG, BG TREND
R squared	0.56	0.84
Absolute error	67.57%	44.57%

Scheme: weka.classifiers.functions.LinearRegression -S 0 -R 1.0E-8 -num-decimal-places 4, Instances: 200, Test mode: 10-fold cross-validation

### Consideration 2.2: Do CDSS consider the sequence of clinical events?

Another consideration is the importance of the sequence that clinical events appear. A case diagnosed with bacteremia (ICD-10: R78.81) followed by severe sepsis (ICD-10: A41.51), which is, in turn followed by septic shock (ICD-10: R65.21) would probably have bad prognosis. The ordered appearance of these three conditions represent a *clinical event* of patient deterioration. A look at the data series below shows Bacteremia and Severe Sepsis diagnoses for 20 patients, their discharge status, and days between diagnosis of Bacteremia and Sepsis, where applicable.

*{Bacteremia (0/1), Sepsis (0/1), Discharge Status (D = dead, A = alive), Sepsis-Bacteremia Days Elapsed}.*

*{0,0, A,} {1,0, A,} {0,0, A,} {1,0, D,} {1,0, A,} {1,1, D,2} {0,0, A,} {1,0, A,} {1,1, D,3} {0,1, A,} {0,0, A,} {0,1, A,} {1,0, A,} {0,1, D,} {1,1 ,A,9} {1,1, D,2} {0,1, A,} {1,1, A,8} {1,1, D,3} {1,1, D,2}.*

For these data, we calculated the conditional probability of death given the existence of Bacteremia to be 50% ([P (D | Bacteremia)] = 6/12 = 50%). The conditional probability of death given the existence of Bacteremia and Severe Sepsis increased, as expected, to 71.4% ([P (D | Severe Sepsis | Bacteremia)] = 5/7 = 71.4%). Next, we used the dichotomous variables ‘Bacteremia’ and ‘Severe Sepsis’ as the only two input variables to predict the risk of death on discharge, with Naïve Bayes, as a base model. The algorithm classified correctly 80% of the instances, with a relative absolute error being equal to 71.95% and a ROC area of 64.8%. After this experiment, we added the third input variable ‘*Sepsis from Bacteremia, Days Elapsed*’. In other words, this variable added to the predictive model, inferred information about the patient response to the antibiotic therapy, prolonging the appearance of Sepsis. Now Naïve Bayes classified correctly 85% of the instances, with a significantly improved absolute error (46.16%) and also an improved ROC area of 75.8%.

### Consideration 2.3: Do CDSS consider the temporal distance of clinical events?

In the aforementioned scenario (2.2), it is also important to consider the temporal distance between the three diagnoses, i.e. how many days after the diagnosis of bacteremia did clinicians diagnose severe sepsis, and then septic shock? Use of timestamps from EMR data in the analysis are important to construct clinically useful events and estimate their severity. This is especially important to assess the performance of health systems in terms of care delivery and transition and eliminate delays and gaps in service.

### Designing systems with contextual validity in mind

#### Core consideration 3: Is the model performance contextually realistic?

When models for clinical predictions are developed, it is essential for health IT designers and data analysts to collaborate closely in order to decide the exact hospital stay phase that the clinical decision will be taking place. This is a very important aspect of correct conceptual designs of data driven clinical CDSS: It is not uncommon that the reported model performance, in various works is overoptimistic, and therefore contextually inaccurate, since the model was trained and tested using input variables which are normally non-available at the point of decision. It is not unusual that published work on clinical predictive models, does not detail the intended use scenario in its methods. When developing predictive models, it is crucial to be aware that a model with high precision and recall in the experimental setup, is not necessarily highly valid in a real context [53].The example of predicting the hospital length of stay (LOS) has been recently explored in many studies [54]. Predicting the hospital LOS is an extremely difficult problem to solve for a patient few hours after admission [55]. This is true since the available patient information at that decision point is only limited the admission information and the patient demographics. The diagnosis, any clinical procedures or medications are still unknown. Few days after the admission, though, predicting the hospital LOS becomes an easier problem, since many more clinical variables became known, contributing to significantly improved performance [56]. Our comparisons in recent work [57] have validated this hypothesis.

### Consideration 3.1: Does the CDSS model the care process on the fly, per user inquiry?

In a real context, training and testing of a model should be repeated dynamically according to the feature-set that varies in different phases of a hospital stay. Each time there is a new inquiry by the user, a new model can be trained, by using only those features which are available at that decision point. A necessary follow-up step would be the testing phase, to establish the degree to which the prediction is contextually valid and therefore clinically useful. The most important limitation for this ‘on the fly model training’ is the computational cost, resulting to systems providing information with delay, after an inquiry. Clearly, the computational complexity of many advanced data analytics methods (e.g. Support Vector Machines, Neural Networks) renders models virtually impossible to be trained on the fly [58]. The clinical decision maker wants to review the prediction output at the point of care, to make decisions accordingly, and cannot afford to wait for hours to see the output.

This is an oftentimes present a machine learning dilemma, which is the model accuracy vs efficiency, and is especially important in healthcare because of the need for very highly accurate predictions, when at the same time decisions need to be made in a non-delayed manner. For the aforementioned reason, low computational cost classification and regression methods such as Bayesian models and linear regression models can be potentially useful. Bayes models perform well in medical problems, due to the highly probabilistic nature of health and disease, despite their inherent variable independence assumption. Researchers recognize the Bayesian approach to decision-making as being the natural statistical framework for evidence-based medicine, incorporating the degree of associated uncertainty [59].

Evidently, with the above methods it is possible to train and test models in a much more reasonable time-frame, with a possible tradeoff being the lower model performance [60]. Options are limited to such computationally efficient methods, to achieve both realistic predictions (training) and assessment of their clinical value (testing), on the fly, at the point of decision. For the problem of predicting risk for hospital infections, Table 2 compares the external validity between: (a) Retrospective models developed in vitro, and (b) Prospective methods involving training and testing of a new model, every time a request is sent to the system.

**Table 2:** Prediction of risk for nosocomial infection

Day of Hospital Stay and Data availability	Use of models built in vitro with “Day 2” variables	Dynamic training & testing
Day 0: Demographics, admission diagnosis	High reported precision & recall is not realistic: Risky decisions, potential negative impact on patient safety	Precision and recall ~ 70% are realistic: Use predictions with a *lot of caution*
Day 1: Demographics, admission diagnosis, medications, lab results	High in vitro reported precision & recall is not realistic: Risky clinical decisions, negative impact on patient safety	Precision and recall ~ 80% are realistic: Use predictions with *some caution*
Day 2: Demographics, admission diagnosis, medications, more lab results, primary diagnosis	High in vitro reported precision & recall is realistic	Precision & recall ~ 85% are realistic

### Consideration 3.2: Are decision makers informed by the system, on-the-fly, about the confidence of predictions, according to the model performance?

The advertised performance of predictive models should be cautiously assessed [61]. The CDSS should inform decision makers about the positive predictive value of a model, which differs in various phases of medical care. Systems should also consider the functionality to warn decision makers that a prediction may not be possible because of critical variables of care missing. Every time a predictive model is trained and tested on-the-fly, prediction results can be presented to the clinical decision maker if the prediction accuracy is satisfactory, or when the standard error is low, without any reservations. In any other case, clinical decision makers would be presented with a system message such as that *‘The outcome cannot be predicted successfully’*.

### Consideration 3.3: Are appropriate data dimensionality reduction methods being utilized?

To make dynamic, on-the-fly predictions possible, an important consideration for designers of CDSS, is data dimensionality. In healthcare, some of the most important information which holds extremely useful predictive value for a series of clinical outcomes of care, is the patient diagnosis and the clinical procedures. The most recent edition of the International Classification of Diseases (ICD-10-CM) includes more than 69,000 different disease codes, to capture specificity [62]. Models which require numeric input variables, will therefore require transformations of each code to a dummy variable. This would generate enormous, sparse datasets, which would make data mining slow. In these cases, the data analyst can consider two different approaches. The first one is dimensionality reduction with methods such as Principal Component Analysis (PCA) [63]. This approach, though, is not possible in explanatory models, such as coefficient analysis in regression, where the model estimates the actual effect of each predictor, on the outcome of interest. A second approach can be the replacement of the ICD codes with groupings (e.g Clinical Classification Software) which are formed by dividing all possible principal diagnoses. The Diagnosis Related Groups (DRG) have also been used in recent studies [64]. Researchers need to decide if the use of grouping methods affects the model performance significantly of not. In short, they need to weigh the improved computational efficiency against a possibly less accurate model.

### The less obvious problem of historical decision bias

#### Core consideration 4: Is ‘historical decision’ bias taken into consideration in CDSS design?

The concept of ‘historical decision’ bias applies to machine learning and statistical methods based on data which encapsulate historical decisions. Historical decision bias occurs when large historical datasets are used to train predictive models which carry over historical human decision errors (such as a misdiagnosis) of the past. This bias is independent of the model performance (precision, recall, or standard error) and refers to the external validity. In other words, testing of a model can result to a high accuracy, which actually represents an accurate method to replicate wrong human decisions of the past. For the patient symptoms and their primary diagnosis data (**{symptom, diagnosis})**:{cough, flu} *, {dyspnea, COPD}, {cough, flu}, {dyspnea, COPD} *, {dyspnea, COPD}, {cough, flu}.

Based on this data, a rule-based model would be generated as follows:

IF Symptom = cough THEN Diagnosis = flu.

IF Symptom = dyspnea THEN Diagnosis = COPD.

Testing would evaluate the model as exceptional, with a perfect ROC area equal to 1. Two of the historical cases, though (the ones with the asterisk) were misdiagnosed patients, but naturally this information is not annotated into the data. If the above model was used for decision support in a real context, it would be mimicking the misdiagnosis decisions of the historical cases, despite the notably impressive model performance. Because of the ‘historical decision’ bias, it is not a good practice to develop therapy recommender systems, solely relying on the patient diagnosis. Considering that historical data include variations of practice, many clinical decisions are not optimal. Similarly, by using past prescriptions as an evidence to select therapies, systems carry this practice variation and decision uncertainty over their current patients [65].

### Consideration 4.1: Are CDSS outcomes driven?

**Fig. 2:**
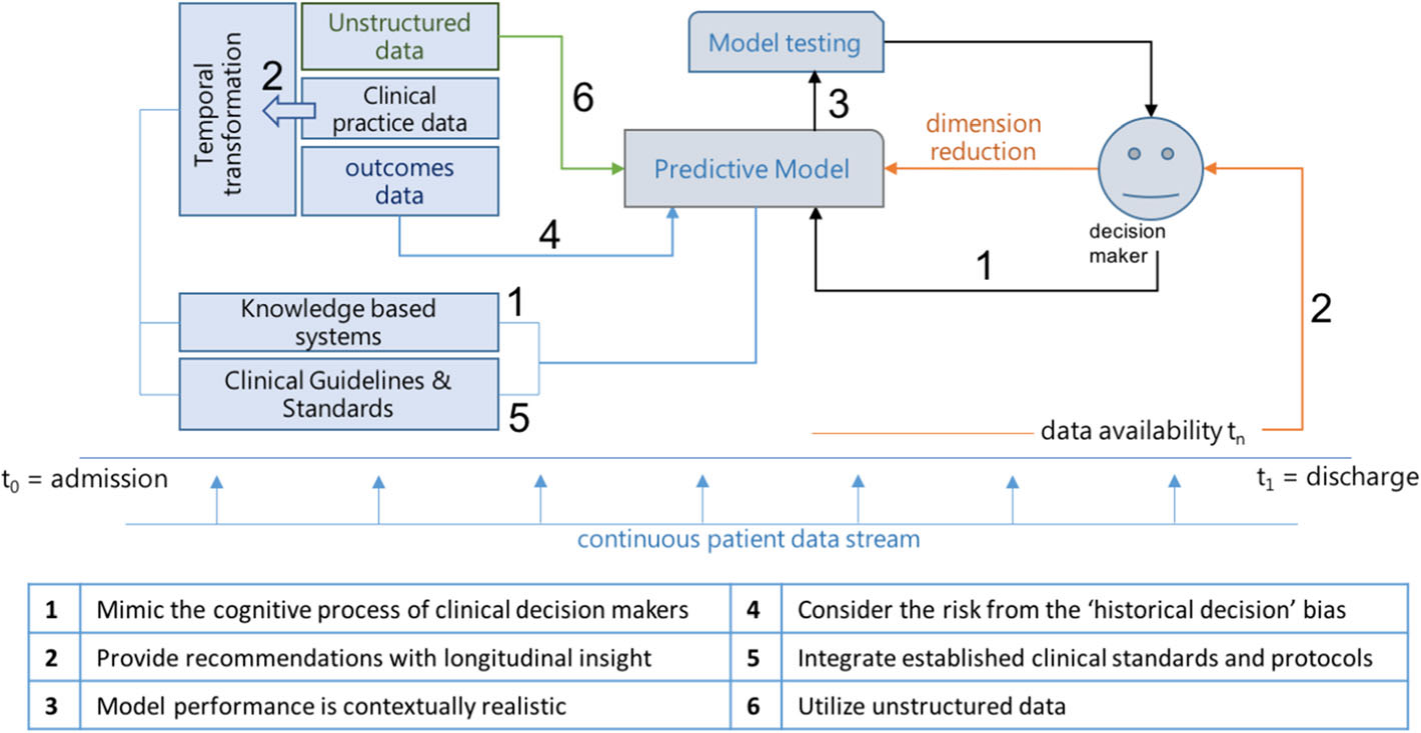
Illustration of the Clinical Decision Support System Reference Model (CDSS-RM)

A useful strategy to eliminate the historical decision bias is to develop methods for outcomes-based predictions. Clearly health professionals such as clinical nursing leaders need to master the skillsets such as the ability to perform outcomes based decision making [66]. Clinical outcomes do not include human subjectivity and can be utilized to select treatments. Take, for instance, a predictive model to assist physicians choose therapy for a patient. The therapy of choice would not be the one that was prescribed to the majority of similar past patients. Instead, it would be that therapy which improved the condition of similar patients in the past (Fig. 2). Such positive clinical outcomes of care can be a hospital discharge without prolonged length of stay, patients not experiencing hospital complications, no records of 30-day unneeded hospital readmissions, and other [67].

### CDSS are designed around established clinical standards and protocols

#### Core consideration 5: Are the de-facto interactions between clinical variables of care modeled according to clinical standards of care?

The established clinical knowledge, clinical guidelines and standards of care direct physicians to specific considerations and use of information during clinical decisions. If portion of the required information is missing, then it may be unsafe to make a decision. An example can be the diagnosis of multiple sclerosis. Diagnostic criteria for this condition include the existence of a combination of central nervous system attacks, lesions, dissemination in space and/or symptom flare ups [68]. These are essential and often mandatory considerations for clinicians to diagnose the condition. In addition, in order to diagnose a condition, physicians review laboratory test results, physiological examination information, the patient history and symptoms [69]. The combination of a symptom of weakness with a low platelet count, are used together diagnose a possible anemia [70]. Since clinically useful information comes from the combination of a multitude of different data, predictive methods can annotate such attributes and their relationship to clinical outcomes. If one of these variables is missing, data scientists should be aware that the predictive value of that model would probably be low. In the example of diagnosing leukemia, both the variables *‘blood cell count’* and *‘bone marrow test’* need to be available to correctly model predictors of leukemia, according to established diagnostic criteria. A high blood cell count, and positive marrow test need to be verified together to diagnose leukemia. Designing CDSS requires this clinical knowledge, and therefore collaboration with health care scientists, as domain experts.

On a side note, knowing the pieces of patient data that need to be evaluated together, can be used as a means of semi-automated feature selection. In typical machine learning approaches, there are two feature selection strategies: The manual feature selection, where the data scientist works together with clinical experts to identify relevant and clinically useful input variables, and the auto feature selection, using exhaustive, best-first and other machine learning approaches. By labelling compulsory feature dependencies, the algorithm ensures inclusion of features that ‘need’ to be input variables, given an outcome of interest, in a semi-automated feature selection approach [71].

### Unstructured health data: An underutilized source of invaluable information

#### Consideration 6: *Does the CDSS utilize unstructured data?*

Nearly 80% of the data in clinical care documents in the U.S is unstructured [72]. These data include free-form files, written physician’s notes, scanned documents, and images. Human to human interactions that can be nurse to physician reporting, patient to physician/nurse complaints and requests, and more, often generate data that, can be stored in an unstructured manner. Unlike structured data which is easy to use, this data is unorganized, text-heavy and hard to process. Natural Language Processing (NLP) methods have been utilized in healthcare research. For example, according to analysis of Electronic Medical Records from emergency departments using text mining of prior expert treatment was shown to provide physicians on call with an optimized treatment plan [73]. Another study, conducted at the University of Utah [74] demonstrated the potential of using NLP systems to automate data extraction. Data extraction, would therefore enrich predictive models with variables and therefore increasing the sensitivity and specificity of algorithms.

## Discussion

The six elements of the CDSS-RM have been introduced and analyzed, in the form of considerations and questions that CDSS designers need to take into account. In an effort to summarize the key points, we provide to the reader, on Table 3 below, the connection of each element to the underlying driving force/decision-making principle, and the consequent derivative design decisions for each one of the elements.

**Table 3:** Relevance of the six CDSS-RM elements and their related CDSS design considerations

Decision Making Principles	CDSS-RM Elements	CDSS Design Derivatives
Health data become useful when combined with human knowledge and experience	1. CDSS mimic the cognitive process of clinical decision makers	(a) Expert systems can be harmonically combined with machine learning (b) Predictive models need to be interactive and react to new info & feedback from clinicians
Clinicians look for changes over time rather than raw measurement values	2. CDSS providing recommendations with longitudinal insight	(a) Models need to include, as predictors, trends of repeated measurements (b) The sequencial order of clinical events should be modelled (c) The temporal distance between clinical events need to be modelled
Data availability varies in different decision points. Data is used accordingly with varying degrees of certainty	3. Contextually realistic model performance	(a) Up-to-date, on the fly training and testing (b) Appropriate dimensionality reduction methods
Copying wrong decisions of historical data is not a good practice	4. ‘Historical decision’ bias is taken into consideration in CDSS design	Design approaches that are built around health outcomes
Data are used according to clinical standards & protocols	5. CDSS integrating established clinical standards & protocols	Models annotate a-priori known variables, in a semi-automated feature selection approach
A significant portion of hospital data are in non-structured formant	6. CDSS utilize unstructured data to enhance feature-set with more input variables for improved performance	Natural Language Processing methods, such as text mining

**Fig. 3:**
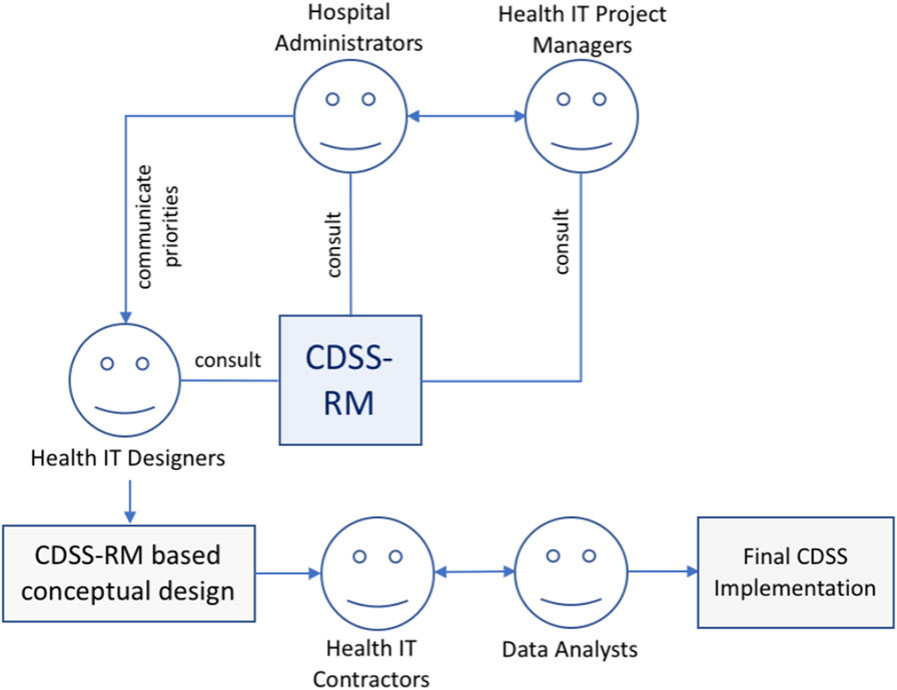
Use-case scenario of CDSS-RM during a CDSS Implementation

This CDSS-RM framework is an effort to articulate in a systematic manner the aforementioned considerations. Its intended use therefore involves health IT researchers, health systems improvement analysts, and IT project leaders, considering the six elements as important priority areas in every CDSS implementation effort. The paper is also anticipated to be useful to the new wave of healthcare administrations who demonstrate a high-level understanding of data analytics: They will learn about, and communicate important design considerations with sub-contracting companies and IT experts, thus bridging an evident communication gap between healthcare and technology. Fig. 3 illustrates an example scenario of a Computerized CDSS implementation, and positions CDSS-RM within multidisciplinary interactions of individuals who consult the reference model.

Figure 2 illustrates, on a diagram, the CDSS-RM model. The six elements have been placed around the typical functionality of a CDSS, during the healthcare services provision, aligned with the temporal aspect of hospital care. With this representation, we attempt a composition of the six elements to give emphasis to the fact that these should not be independently assessed, but within the context of the continuum of care.

Data scientists want to have rich data at their disposal. Utilizing historical data from Electronic Medical Records, which typically store every detail of the clinical care, is foundational for successful models [75]. Although not always feasible due to data privacy restrictions, these data sources are preferred over external de-identified datasets. Such datasets, mainly available from the Centers for Medicare and Medicaid Services (CMS) in the United States, include limited number of clinical variables and are de-identified. While useful in health quality research, these datasets should not be used for an accurate modeling of the clinical care process but are more oriented towards quality assessment research.

Methods developing CDSS are founded upon the unique nature of the use and flow of health information. It is critical that CDSS simulate the cognition of clinical decision makers; health data become useful when combined with human knowledge and experience. Since health data are assessed by health professionals who process information with their cognitive skills, design approaches combining expert systems with the power of machine learning can increase the clinical value of CDSS. Predictive models need to be dynamic and re-estimate predictions according to new information and feedback from clinicians, while providing recommendations with a longitudinal insight. Any decision support method needs to consider trends and changes of the physiological measurements. Such trends of repeated measurements, rather than raw values are more suitable predictors of health outcomes. In addition, CDSS should be ‘self-aware’ of their use-context: In different decision time points, a pre-fabricated algorithm would output predictions often externally invalid, due to varying data availability.

With respect to the above, CDSS should also provide dynamic predictions by interacting with decision makers, re-estimating predictions according to new clinical information or to reinforced feedback. Updated re-trained and re-tested dynamic models, can provide up-to date and therefore contextually relevant information. Decision makers should be cautious with overoptimistic in vitro model performance reports.

The ‘historical decision’ bias that was discussed in this paper, should be avoided, so far as possible. It is not a good practice to perpetuate medical mistakes, such as misdiagnoses, and non-optimal prescriptions, which are evidently included in historical datasets. Decisions should not just replicate historical patterns of care, but should also be driven by those historical practices leading to desirable clinical outcomes. Using outcomes-based approaches, when designing CDSS is recognized as a good practice. CDSS should also model any a-priory known interactions between clinical attributes and recognize variables which are evidently used together in decision-making. CDSS designers can therefore annotate such groups of variables to co-exist as predictors. Dimensionality reduction, finally, can improve model efficiency and facilitate on-the-fly training of models. Dimensionality reduction can be statistical (e.g. Principal Component Analysis) or non-statistical (e.g. regrouping ICD-10 diagnoses).

With the above considerations in mind, CDSS can be optimized, become healthcare-decision-making relevant, and therefore more useful in the real hospital context, to address critical decision-making challenges. Such systems will provide evidence based recommendations to clinicians to improve their capacity and their insights, in an effort to achieve high quality and safe service, adding value to health organizations.

### Limitations

While this work provides a solid framework of important aspects for conceptual designs of CDSS systems, it is not applicable to every health environment. The framework defines the clinical decision making requirements in a clinical hospital environment. It therefore does not address structural, and infrastructural aspects of health organizations. For instance, it does not account for aspects of continuum of care and infrastructure limitations, such as lack of standards and interoperability. The intended scope of this framework precedes technical implementation approaches and serves as an aid to understand aspects related to proper selection of variables and data, and relevant designs of algorithms. While the authors present the CDSS-RM components in a structured way, and based on literature knowledge and their field experience, the acknowledge that other considerations, not discussed in this paper, could also add value to conceptual designs. The authors, finally, believe that further work can focus on connections between the framework components and implementation aspects, such as the use of temporal algorithms (e.g. temporal Bayesian networks) and dynamic classification methods, and evaluation and validation of the model with feedback from actual clinical care providers and clinical decision makers.

## Conclusion

This paper introduces a reference model for Clinical Decision Support System design, with six elements (CDSS-RM), and connections of each element to the underlying driving force/decision-making principle. The six elements are placed around the typical functionality of a CDSS, during the healthcare service, aligned with the temporal aspect of hospital care, and within the context of the continuum of care. It is critical that CDSS simulate the cognition of clinical decision makers, since health data become useful when combined with human knowledge and experience. Design approaches combining expert systems with the power of machine learning can increase the clinical value of CDSS. Predictive models need to be dynamic, re-estimating predictions according to new information and feedback from clinicians, while providing recommendations with a longitudinal insight. Any decision support method needs to consider trends and changes of the physiological measurements, as these are more suitable predictors of health outcomes, than cross sectional values. In addition, CDSS should be ‘self-aware’ of their use-context: In different decision time points, a pre-fabricated algorithm would output predictions often externally invalid, due to varying information availability and decision makers should be cautious with overoptimistic in vitro model performance reports. With respect to the above, CDSS should provide dynamic predictions, and interact with decision makers to re-estimate predictions according to new clinical information. The ‘historical decision’ bias should be avoided, so far as possible. It is not a good practice to perpetuate medical mistakes, such as misdiagnoses, and non-optimal prescriptions, which are evidently included in historical datasets. Decisions should not just replicate historical patterns of care, but should also be driven by those historical practices leading to desirable clinical outcomes. Using outcome-based approaches, when designing CDSS is recognized as a good practice. CDSS should also model any a-priory known interactions between clinical attributes and recognize variables which are evidently used together in decision-making. CDSS designers can therefore annotate such groups of variables to co-exist as predictors. With the above considerations in mind, CDSS can be optimized, become healthcare-decision-making relevant, and therefore more useful in the real hospital context, to address critical decision-making challenges. Such systems will provide evidence based recommendations to clinicians to improve their capacity and their insights, in an effort to achieve high quality and safe service, adding value to health organizations.

## Abbreviations

CDSS: Clinical Decision Support Systems
CMS: Centers for Medicare and Medicaid Services
DRG: Diagnosis Related Groups
ICD: International Classification of Diseases
LOS: Length of Stay
NLP: Natural Language Processing
PCA: Principal Component Analysis
TITE: Time-Interactions-Trends-Events
